# Building Climate Resilience in Health Systems: A Climate Vulnerability and Capacity Assessment in a rural hospital in Chad

**DOI:** 10.5334/aogh.4743

**Published:** 2025-08-19

**Authors:** Patricia Nayna Schwerdtle, Didier Tokoumnogo Zidouemba, Alexi Reouhiri Dermbaye, Kiran Jobanputra, Melissa Mcrae, Melanie Tarabbo, Mohamed Njouonkou, Marius Madjissem, Alexandre Robert, Zia Haider

**Affiliations:** 1 Heidelberg Institute of Global Health, Heidelberg, Germany; 2 Interdisziplinäres Zentrum für wissens, Germany; 3 The Climate Action Accelerator, Geneva, Switzerland; 4 ALIMA, Ngouri, Chad; 5 Alerte Sante, Ngouri, Chad; 6 Ministère de la santé publique, Hôpital de District de Ngouri, Ngouri, Chad

**Keywords:** climate change and health, climate resilience, climate change adaptation, climate vulnerability, adaptive capacity, environmental sustainability

## Abstract

*Background:* Chad is highly vulnerable to climate change, posing significant threats to health systems and population health. Rising temperatures, irregular rainfall, droughts, and resource scarcity exacerbate food insecurity, malnutrition, and vector-borne diseases like malaria. In Ngouri, a rural area in the Lac Region, these climate stressors have led to worsening health outcomes and strained healthcare services. Without adaptation measures, facilities will struggle to maintain essential services amid escalating climate pressures. This case study presents a facility-adapted climate vulnerability and capacity assessment (VCA) for a rural hospital in Chad, identifying key risks and prioritizing solutions to enhance climate resilience.

*Objectives:* This case study describes the development and implementation of a facility-adapted climate VCA in a high-vulnerability, low-resource setting and outlines prioritized solutions for an actionable adaptation plan.

*Methods:* The study employed a participatory mixed-methods design, incorporating five stages: (1) literature review on climate hazards, exposure pathways, and population vulnerabilities; (2) facility audit assessing infrastructure and healthcare delivery gaps; (3) qualitative focus groups to refine risk identification; (4) development of a matrix of solutions with cost estimates and feasibility analysis; and (5) a participatory prioritization process to develop a multi-year facility improvement plan.

*Findings:* The VCA identified climate risks, including elevated malarial mortality and power outages disrupting oxygen supply. A list of 35 solutions was generated, with 22 priority actions selected for implementation. These included anticipatory planning, community sensitization, supplementary feeding programs, and improved waste management. The process highlighted the importance of community engagement, multidisciplinary collaboration, and staff motivation for climate-resilient and sustainable healthcare.

*Conclusion:* The adapted VCA offers a replicable approach to assessing climate-related vulnerabilities and capacities in healthcare facilities. It revealed significant risks to health service delivery and informed the development of locally feasible, sustainable adaptation measures. The framework applied in Ngouri hospital demonstrates relevance for similarly affected contexts, supporting efforts to align health systems with global sustainability goals and to secure quality healthcare under changing climatic conditions.

## Background

### Climate change impacts in chad

Chad, located in the Sahel region of Africa, is highly vulnerable to the current and projected impacts of climate change [1, 2]. The country already faces significant challenges, including recurrent droughts, desertification, and food insecurity, which are expected to worsen with climate change [3–5]. The current impacts of climate change in Chad include increased temperatures, irregular rainfall patterns, and prolonged dry seasons, leading to reduced agricultural productivity, water scarcity, and loss of natural resources [6–8]. These factors contribute to food insecurity, malnutrition, and displacement of populations [9, 10]. Additionally, climate change exacerbates existing social and economic vulnerabilities [11], increases the risk of conflicts over scarce resources [12], and poses significant health risks [13], including the spread of vector-borne diseases [14, 15] and heat-related illnesses [16, 17].

Ngouri, in the Lac Region in southern Chad, is particularly affected by the impacts of climate change [18]. It experiences high temperatures and decreased rainfall, resulting in water scarcity and reduced agricultural output. The region faces challenges such as food insecurity, malnutrition, and increased vulnerability to waterborne and vector-borne diseases [19]. As climate change continues, the projected impacts in Ngouri include further reductions in crop yields, changing patterns of infectious disease outbreaks including malaria, increased frequency and intensity of droughts and heatwaves, and the potential for increased conflicts over limited resources. These changes will have direct impacts on population health (e.g. increased heat-related exacerbations of chronic disease) as well as indirect impacts such as malnutrition due to food insecurity [20], increased mortality due to increased incidence of severe malaria [4], and overwhelming pressure on hospital services [6, 21]. These added and exacerbated shocks and stresses on health facilities require resilience-building activities so that services can be strengthened and continue.

Given the high incidence of malnutrition and malaria in this region, and the fact that these are the highest priority conditions for Ngouri hospital (see below), we briefly outline the links between these two health issues and climate change. Climate change will change patterns of infectious diseases like malaria. Rising temperatures and altered precipitation patterns create favorable conditions for the proliferation of malaria-carrying mosquitoes [22–24]. As temperature and humidity rise, mosquitoes expand into new regions, exposing previously unaffected populations. Changes in rainfall patterns can also create more breeding, increasing the risk of malaria transmission [25, 26]. These environmental shifts can lead to higher mosquito densities and longer transmission periods, further heightening the risk.

Additionally, climate change plays a significant role in malnutrition. Altered weather patterns can affect crop yields and reduce the nutritional quality of food, leading to food insecurity [27–29]. Extreme weather events, such as droughts and floods, can disrupt agricultural production and compromise access to nutritious food sources [30]. The resulting decline in food availability and quality contributes to malnutrition, particularly in vulnerable communities that rely heavily on subsistence agriculture [17].

### Importance of climate resilience in healthcare facilities

Climate change resilience is important for health facilities and systems as they increasingly face various climate-related shocks and stresses [31]. Climate resilience is particularly important in resource-limited settings where health systems can be fragile and alternative options for care are limited or non-existent. Health facilities need to be prepared to address the evolving challenges posed by climate change to ensure the continuity of care and safeguard the health and well-being of populations [32]. Climate change can directly impact health facilities through extreme weather events, such as floods and storms, which can damage infrastructure, disrupt services, and compromise the safety of patients and healthcare workers. Additionally, climate change can indirectly affect health facilities by influencing disease patterns, exacerbating the spread of vector-borne diseases [25, 33], increasing the prevalence of heat-related illnesses [16, 17], and impacting water and food security [34, 35]. By building climate change resilience, health facilities can enhance their capacity to withstand and respond to these challenges, ensuring uninterrupted access to quality healthcare services, protecting the health workforce, and providing effective emergency response in response to climate-related disasters. Furthermore, climate change resilience in health facilities can contribute to broader community resilience and adaptation efforts, promoting sustainable and equitable health systems that are better prepared to address the health impacts of a changing climate and other threats.

## Literature Review

As climate change accelerates, the health sector is more exposed to climate-related threats; however, it is also complicit in the problem, by contributing around 4.4% to global GHG emissions, primarily through supply chains, transportation, heating, ventilation, and waste management [36–38]. The healthcare sector is globally challenged by climate change and environmental threats, adding to the burden faced by countries as they strive to provide effective, efficient, and equitable healthcare. Low-income countries, those with pronounced inequities, and those characterized by fragile institutions and weak governance are particularly challenged [34, 39, 40]. These settings are more likely to be highly vulnerable with low readiness, and they are more likely to have weaker health systems, to be further off universal healthcare, and tend to carry higher burdens of disease [8, 34, 40]. A recent scoping review of climate-resilient health systems in LMICs found that climate change will lead to damaged infrastructure, more patients, and a stressed workforce [41]. To address this complex challenge, there is an urgent need to adopt climate-resilient, sustainable, low-carbon healthcare system models, and this aligns with the Sustainable Development Goals (SDGs) and the Paris Agreement targets [42–44].

We reviewed a range of tools available for conducting vulnerability and adaptation assessments at the national, health systems and health facility levels. We started with the “WHO checklists to assess vulnerabilities in health care facilities in the context of climate change” [45] and the “WHO guidance for climate resilient and environmentally sustainable health care facilities” [46]. We observed that they were designed to be used together, so we created a new mixed-methods methodology to combine the most useful elements of these two key resources to align with the practical purposes of our project. In the course of applying this methodology, we brought in elements of a third resource, the “WHO operational framework for climate resilient and low carbon health systems” [47], to address systems issues that play out at the level of the facility [47– 46]. This case study aims first to describe the process of developing and conducting this facility-adapted climate vulnerability and capacity assessment (VCA)^1^ for a hospital in a low-resource setting with high vulnerability and low readiness. Second, we aim to describe the findings as a prioritized list of solutions, identified for the hospital, which will be translated into an actionable adaptation plan.

## Methods

### Setting description

Chad, a landlocked country in northern central Africa, situated within the Sahel region, is facing significant challenges related to climate change. With a population of 18 million people in 2023 and an annual population growth rate of 3%, Chad faces a complex demographic landscape [48]. The majority of its inhabitants reside in the central and southwestern regions around Lake Chad, while the sparsely populated north experiences a hotter and drier desert climate. Classified as one of the world’s poorest countries [49, 50], Chad operates as the least developed country (LDC) [51]. Dominated by agriculture, contributing 44.9% to the GDP in 2018 [6], Chad’s economy is vulnerable and heavily reliant on smallholder farming and rainfed practices for food security and livelihoods. According to the ND Gain Index, Chad is the second most vulnerable country and the 190th most ready country concerning climate change [52]. Climate change poses a significant threat, bringing rising temperatures, reduced water availability, and more frequent floods and extreme weather events. With limited adaptive capacity, Chad faces heightened vulnerability to climate change, further compounded by the country’s history of civil wars, intercommunal conflicts, and forced displacement. This complex context underscores the urgent need to build climate resilience, particularly in health facilities, to mitigate the anticipated health impacts of climate change on Chad’s vulnerable population [6].

Ngouri district is in the Lac region, which is heavily affected by insecurity (land Nigeria, Boko Haram incursion, population displacement, etc.). The district is in the middle of the Sahelian zone, on the edge of the desert, which is advancing steadily. It is an area of high food and nutritional insecurity. An increase in rain and river flooding in recent years has had negative consequences for agricultural production.

**Figure 1:**
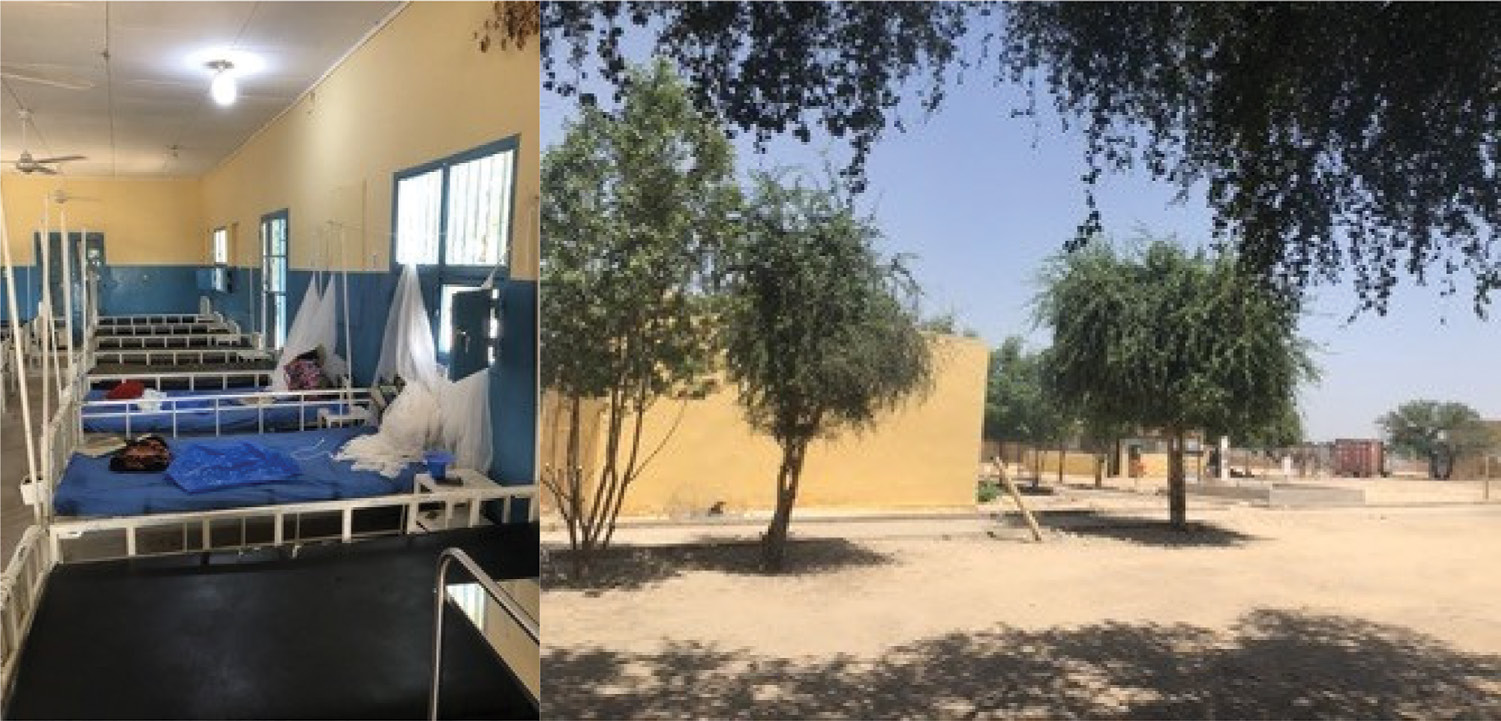
Photos from the hospital: IPD ward and outside the hospital with the Triage building behind the tree on the right.

**Figure 2:**
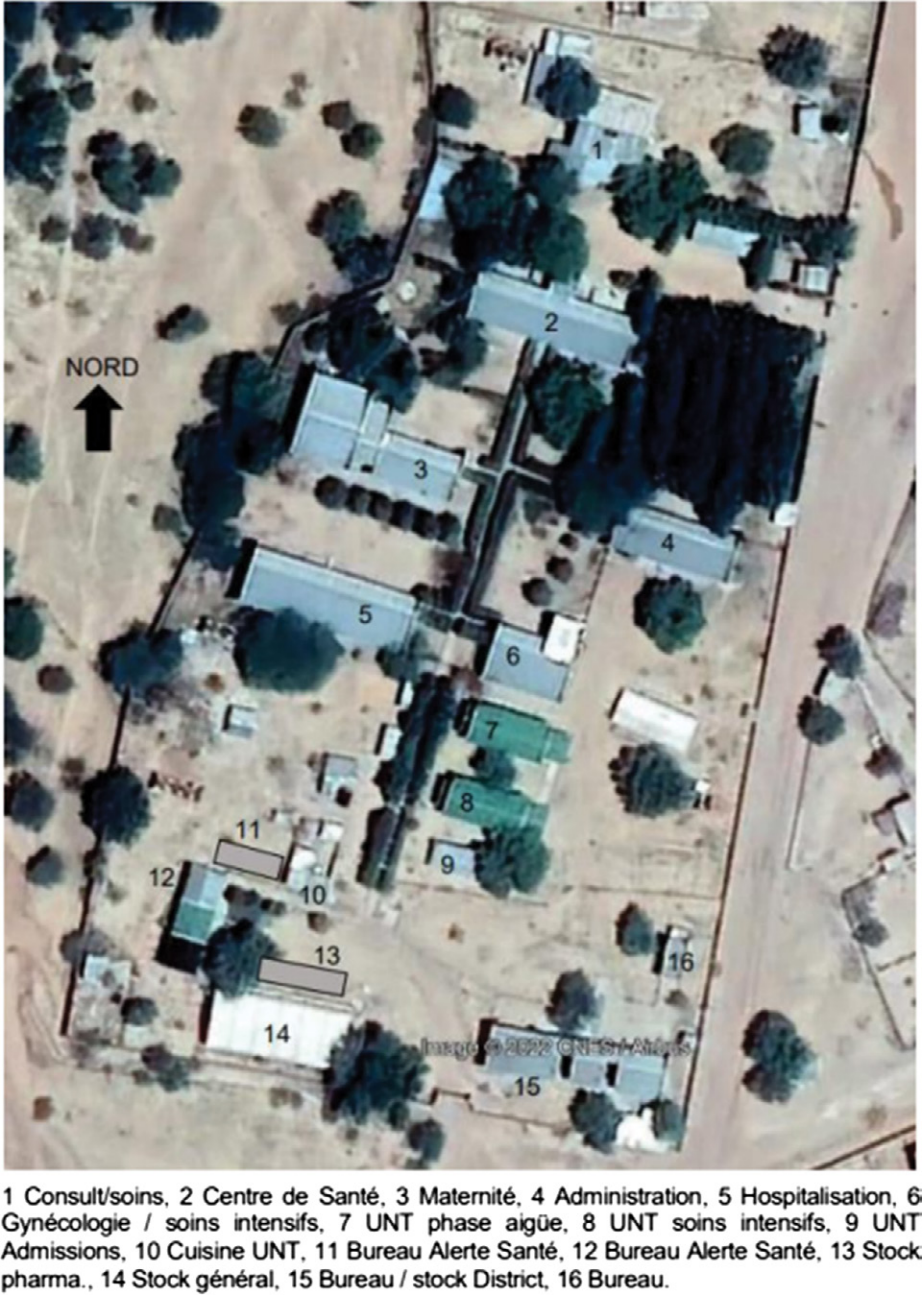
Satellite map of the hospital.

Ngouri hospital is a district general hospital with approximately 100 beds, run by the Chadian Department of Health (DoH) and supported by the Alliance for International Medical Action (ALIMA) and a Chadian medical humanitarian NGO, Alerte, Sante, for over a decade (See Figure 1 and 2). It is a secondary-level referral hospital to 34 health centers that serve a catchment population of approximately 164,000 people. For complex cases, a referral is required to the National Reference General Hospital—*l’Hôpital General de Reference Nationale* in N’djamena. Ngouri hospital is 235 km north of N’djamena, but there is no system of medical transport available for the people in Ngouri. Some patients are supported by ALIMA to travel to the tertiary referral hospital by private car. Within the hospital, ALIMA supports 50 in-patient beds for pediatric patients (aged mostly under 5 years), and an ICU (with oxygen therapy and intensive care). The rest of the hospital includes maternity and gynecological services, surgery, TB care, and an outpatient service with two consultation rooms. During peak times, the hospital facilities are routinely overwhelmed and there was a need to set up a large tent in the hospital courtyard to accommodate patients, mainly children, requiring treatment for malnutrition and severe malaria. Extreme heat and severe dust make working and treatment conditions for staff and patients extremely difficult. Currently, the hospital has sufficient water from the borehole; however, there are concerns about future supply. Grid electricity supply from the city is erratic, such that the hospital has to generate its own electricity. Solar infrastructure is currently limited, and so although lighting and fans run on solar energy, critical services such as vaccine fridges and sterilization equipment are all on diesel generator backup. Due to the heat within the hospital buildings, beds are often empty during the day and patients are treated under the trees in the courtyard.

ALIMA works in partnership with another NGO—The Climate Action Accelerator (CAA) to improve the environmental sustainability of its operations. Together with the DoH and Alerte Sante, the four partners agreed to work together on a project to improve the climate change resilience and the environmental sustainability of Ngouri Hospital, establishing a multidisciplinary team to organize this, including representatives from all four agencies, in line with WHO guidance [31, 46, 53]. This multidisciplinary team (henceforth referred to as the Climate Resilient and Environmentally Sustainable Hospital team or “CRESH” Team) consisted of a hospital director, head of logistics, district MoH representative, representatives of the two supporting NGOs (ALIMA and Alerte Sante), senior clinician, and a CAA facilitator.

### Description of how the climate VCA approach was developed

A climate VCA for a health facility is the first step to developing greater climate change resilience. The assessment helps identify the facility’s weaknesses and strengths regarding climate-related risk preparedness and mitigation, enabling tailored strategies to enhance the facility’s preparedness and adaptive capacities against climate-related challenges.

***Vulnerability*.** refers to the tendency or likelihood to be negatively affected by climate change-related hazards. Vulnerability includes having a higher chance of suffering harm and a lack of capacity to cope and adapt when harm occurs [31, 53, 54]. An example of a climate change vulnerability for a health facility could be an increased risk of flooding due to more frequent and intense rainfall events, which could damage infrastructure, disrupt services, and compromise the facility’s ability to provide healthcare during emergencies.

***Adaptive capacity*.** refers to the ability of systems, institutions, humans, and other organisms to adjust to potential damage, take advantage of opportunities, or respond to consequences [55]. For the health workforce, an example of adaptive capacity could be implementing training programs that educate healthcare professionals about climate-related health risks and equip them with the skills to provide effective care during climate-related emergencies, such as heatwaves or disease outbreaks. In terms of health information systems and early warning, adaptive capacity might involve developing real-time monitoring and communication systems that provide timely information about changing climate conditions and related health threats. This could allow the health facility to anticipate and respond to climate-sensitive disease outbreaks or other health risks promptly.

***The VCA process*.** Once the CRESH team agreed to develop an adapted VCA process to be piloted in Ngouri hospital, the key objectives and assumptions for the VCA were established. The primary objective of the assessment (and interventions) is climate change resilience and improving the health of the local population. The secondary focus is assessing (and improving) the environmental sustainability of the facility, including the carbon footprint.

The output of the assessment should be a prioritized list of solutions that can be developed into a comprehensive, multi-year adaptation plan for the hospital. Existing tools were reviewed [31, 41, 47, 45, 48, 25, 38, 45, 46] and informal discussions were held with other agencies working in this domain. An exploratory visit was made to Ngouri to familiarize CAA with the project location and function and to explore the climate awareness and priorities of the hospital staff. Awareness of climate change was found to be high and there was a keenness to improve practices. However, this was constrained by limited knowledge of practice and technical confidence in what would be required to build resilience sustainably. The team concluded that the process would need to be highly inclusive, and included a third objective for the VCA: creating change dynamism for sustainability and resilience.

**Figure 3:**
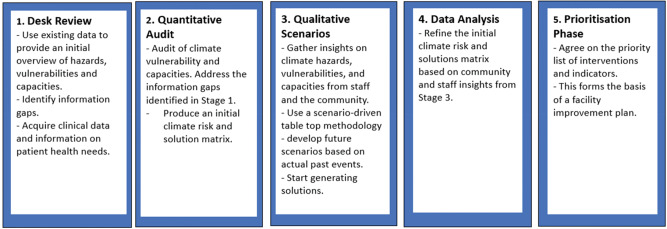
Health facility climate VCA process.

The WHO “Checklists to assess vulnerabilities in healthcare facilities in the context of climate change” and the WHO “Climate resilient and environmentally sustainable healthcare facility guidelines” were used as a basis for the development of a mixed-methods approach to meet these objectives [45, 46] with the following five steps (Figure 3).

### Description of the implementation plan

Within the CRESH team, the ALIMA representative and the CAA facilitator coordinated the process, with the CAA facilitator providing brief training to the rest of the team on the climate VCA approach. The climate VCA was conducted over the first six months of 2023, requiring two weeks of on-site data collection at the hospital.

**In Stage 1:** A rapid “operational” literature review was carried out based on a comprehensive search of the academic and gray literature and unpublished reports and data from Ngouri hospital. This included population health and weather/climate data. No local meteorological data were identified, requiring higher level, regional sources to further elaborate the hazards and exposures (see Annex 3: Climate Information Sources for Stage 1 and 3). This yielded a summary of climate hazards in the Sahel region, together with basic info on population vulnerabilities. It also provided basic logistics information on the hospital facilities, which enabled the narrowing down of the audit to address the information gaps.

**Figure 4:**
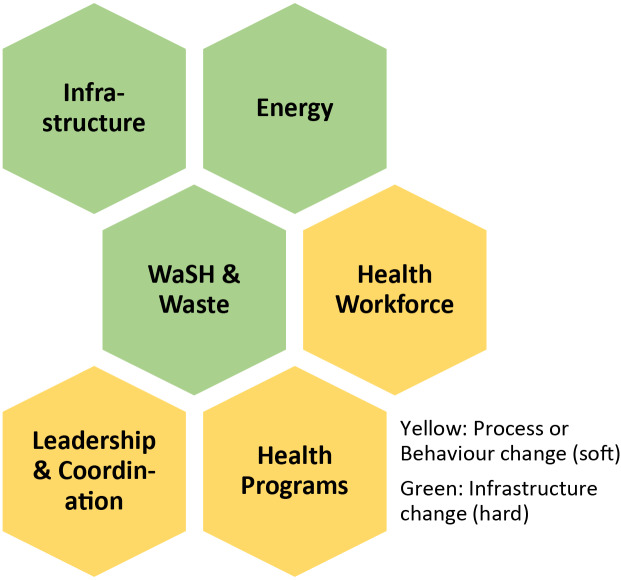
CRESH modular approach [46]. Module 1: Infrastructure, technologies and products. Module 2: Energy. Module 3: Water, sanitation, hygiene and healthcare waste. Module 4: Health workforce. Module 5: Health programming. Module 6: Leadership and coordination.

**In Stage 2:** The facility audit takes a classic checklist approach and involves a walk-through of the hospital, drawing on the knowledge and skills of data, logistics, and health staff. The audit was developed before the visit and focused on six modules (see Figure 4). The audit highlighted infrastructural vulnerabilities and opportunities for strengthening workforce management and aspects of healthcare delivery. The emerging information was integrated into the climate health risk and solution matrix.

**In Stage 3:** Facilitators were trained in a workshop beforehand, supported by an anthropologist who gave different options on how to capture the outputs. Initially, four focus groups were planned: (1) healthcare workers, (2) health administrators, (3) community leaders, and (4) patients and relatives. Focus groups also included several groups of people from the Ngouri locality, including the village chief, health center managers, and leaders of women’s groups. In the end, groups 3 and 4 were combined but then separated by gender, which was thought to be important to elicit the contributions of all participants. A storytelling approach was used, combined with a tabletop methodology whereby a visual aid (map on a table) prompted participants to talk through how they experienced a climate hazard. Participants were asked to begin when they woke up and use the visual aid to lead them through the day. With the aid of a facilitator, the first person tells their experiences and then other participants are invited to tell their own version, but also to draw out common experiences or differences and understand why they remember aspects differently. The output of the conversation was a tree diagram that captured the key climate hazards (roots) and the impacts (branches: yellow) and possible solutions to mitigate risks (branches: green) (see Annex 2: Tree Diagram). The qualitative work generated an accurate example of the most important risk for the population of Ngouri—malaria outbreak, showing the interaction of health facility vulnerabilities, capacities, and potential solutions. This enabled the enriching of the “climate health risk and solution matrix,” and helped generate a preliminary list of climate risks and interventions to address these hazards.

**In Stage 4:** The climate health risk and solution matrix could now be fully elaborated. The initial list of solutions generated through the qualitative work was expanded using CAA’s generic solutions list,^2^ and through discussions with technical experts (architect, energy, biomedical and WaSH) within CAA. Parameters to enable prioritization were agreed with the CRESH team (such as cost and visibility of interventions), and data were added with regard to these parameters relevant to decision-making.

**In Stage 5:** The climate health risk and solution matrix was reviewed on a preliminary basis by the CRESH team, with input from colleagues in the Ministry of Health, who excluded any solutions that were not feasible, already implemented, or inconsistent with the values of the hospital and supporting partner (ALIMA). Further information was added (on security/access) to enable decision-making. A formal prioritization workshop was then held for the full CRESH team to review and prioritize the identified interventions and to produce a preliminary shortlist to propose to senior managers in ALIMA. A second workshop was organized involving both the CRESH team and the senior managers of ALIMA, at which the proposed shortlist was further examined, modified, and finally approved. This finalized matrix was then used to develop a multi-year facility improvement plan (with detailed activities, indicators, and indicative budget), from which funding proposals for individual interventions will be derived.

**Ethical considerations:** The climate VCA was developed as an operational tool through a collaboration between the Ministry of Health, ALIMA, Alerte Sante, and CAA, in consultation with WHO and making use of available literature and guidance on the topic. This descriptive study builds on data collected through the process of development and implementation of the VCA tools and requires additional data collection beyond data that was already collected for operational purposes. The study was conducted with permission from the Ngouri prefectural health authority and the Chadian Ministry of Health.

## Findings

### Assessment of the hospital’s vulnerabilities and capacity to deal with climate change impacts

The VCA enabled the team to produce a climate change risk register for the hospital—essentially a spreadsheet detailing the specific (i) climate change hazards, (ii) exposures, (iii) vulnerabilities and adaptive capacities, (iv) health risks, for the population and facility, and (v) potential mitigation interventions (solutions) for Ngouri hospital.

The main hazards identified for Ngouri were (i) warming temperatures, deforestation, changes in rainfall pattern, and water source distribution, leading to changes in infectious disease epidemiology; (ii) drought and heatwaves; (iii) floods and storms; (iv) other hazards of concern, including the cost-of-living crisis, pandemics, insecurity, conflict, and terrorism.

The main risks identified were grouped into three domains: direct and indirect health impacts, impacts on the facility and the health workforce. First, direct and indirect health impacts included changing patterns of climate-sensitive diseases (like malaria and meningitis). Increased infectious disease mortality emerged as the greatest risk, primarily due to malaria. This risk explains the emphasis on solutions like (1) energy interventions to stabilize the oxygen supply; (2) reinforcing the blood bank; (2) indoor residual sprays (IRS) and bed nets; and (4) supplementary feeding to prevent malnutrition. Other health impacts of concern included increasing levels of malnutrition, and rising poverty and displacement. Second, climate impacts on the facility, such as high-temperature exposure, insufficient reserves in the blood bank, and medication supply shortages due to overconsumption and interrupted supply. Third, impacts related to the health workforce include burnout due to excessive workload, extremely hot working conditions, and stress from dealing with high mortality rates among the patient population.

Interventions to mitigate the risks identified above included: anticipatory systems and seasonal calendars to plan buffer stocks and hospital workforce deployment, an affordable referral and transport system, community sensitization to climate-related health impacts current and anticipated, supplementary feeding program three months before anticipated malaria/malnutrition peaks, routine community surveillance, and improved oxygen management and waste management activities. In the next section, we describe the list of proposed interventions to build climate resilience with potential indicators to monitor and evaluate implementation.

### A final prioritized list of interventions to form the basis of the adaptation plan

A list of 35 potential solutions was generated at step 4 (Risk-solution matrix) of the VCA process (see Annex 1). After the prioritization workshop (step 5), the list of solutions was reduced to 22; nine structural solutions (solutions 1–9) and 11 process solutions (10–22). Note, the solutions do not align perfectly with the risk because some risks correspond to multiple solutions, and some solutions address multiple risks (Table 1). See Box 1 for a brief explanation of the six categories.

**Table 1:** Prioritized climate solutions.

SOLUTION	CATEGORY	DESCRIPTION
1	Infrastructure, technology, and products	Additional multi-functional structure to increase the reception capacity of peaks
2	Infrastructure, technology, and products	Renovate/repaint internal hospital surfaces to improve infection control
3	Infrastructure, technology, and products	Reduce overall indoor temperatures with reflective paint on roofs
4	Infrastructure, technology, and products	Improve ventilation through ventilation chimneys and slatted windows/shutters
5	Infrastructure, technology, and products	Improve O_2_ management to ensure resilience in the case of peaks (efficient concentrators/bridging system)
6	Energy	Improve energy infrastructure to ensure resilience and stabilize the ICU/ pharmacy temperature; solar system expansion; upgrade battery housing with suitable bridging system; motion sensors to reduce lighting usage
7	Energy	Develop and implement an energy management protocol to reduce consumption
8	Wash and waste	Strengthen medical waste infrastructure through a communal waste zone
9	Wash and waste	Assess the possibilities for recycling waste (e.g., plastics) in Ndjamena
10	Workforce	Implement a hospital Hygiene Committee (entire hospital)
11	Workforce	Staff training in self-care and sustainable healthcare, energy management, waste management, effects of heat on patients, modification of ID distribution, etc.
12	Workforce	Promote or develop a disaster management committee at the local/hospital level
13	Health services	Reinforce community sensitization activities around malaria, heat, non-communicable diseases, diarrhea, etc.
14	Health services	Strengthen the blood bank, including raising awareness for donor recruitment and providing testing reagents
15	Health services	Extend the current (free) ambulance service (including boat transfer) for children under 5, to include older children and pregnant women
16	Health services	A package of preventative nutritional interventions (including nutri-vacc) 3 m before the peak of malaria
17	Health services	Review and realign vaccination, malaria, and malnutrition prevention activities to reduce missed opportunities
18	Health services	Distribution of mosquito nets and indoor residual insecticide spraying.
19	Governance and financing	Implement a seasonal risk calendar to help hospital leadership anticipate needs and plan buffer stocks accordingly.
20	Governance and financing	Review and strengthen facility emergency plans (EPREP) to cover all risks, including climate and pandemic risks.
21	Governance and financing	Rigorously monitor and evaluate implementation to adjust activities and demonstrate “best buys”
22	Governance and financing	Integrate this project into national (or organizational) action plans and roadmaps to ensure sustainable funding and increase scalability.

**Box 1: A climate-resilient and environmentally sustainable health facility incorporates the following six components**

**Infrastructure, technology, and products:** Climate-resilient building designs that are resilient to extreme weather events and conditions. Health facilities with energy-efficient equipment and materials that reduce resource use and waste.

**Energy:** Sustainable energy systems that provide adequate, reliable, and renewable power sources (such as solar or wind energy) that reduce dependency on fossil fuels and enhance resilience during power disruptions.

**WASH (Water, Sanitation, and Hygiene) and Waste:** Effective water management, sanitation systems, and sustainable waste management practices. A safe water supply, eco-friendly sanitation facilities, and minimizing hazardous waste through recycling and proper disposal methods.

**Health workforce:** A climate-resilient workforce is trained to respond to climate impacts on health, is knowledgeable about environmental sustainability practices, and can adapt services and operations to maintain health services during climate-related challenges.

**Health services:** Climate-resilient health services focus on maintaining continuity of care despite climate hazards and disasters and adjust to the changing burden of climate-sensitive diseases. This includes strengthening early warning systems, surveillance, and response mechanisms for climate-induced health risks.

**Governance and financing:** Effective governance and financing mechanisms support the integration of climate resilience and sustainability into health policies, operational plans, and budgets. This includes securing funding for sustainability initiatives, ensuring policy alignment, and fostering cross-sector collaboration for comprehensive climate action.

Based on the WHO Operational Framework for Climate Resilient and Low Carbon Health Systems and the CAA VCA Toolkit.

### Successes and challenges faced during the VCA implementation

The full VCA took several months; however, step 3 (qualitative scenario phase) took five days. During these five days, participants were satisfied with the quality of the exchanges and expressed high hopes of seeing the Ngouri hospital become resilient to the impacts of climate change. They valued the inclusion of the broad perspectives of many partners at different levels, from care providers to community leaders. There was good participation in not only identifying vulnerabilities but also proposing solutions. The process enabled the team to appreciate current and future threats that many had not connected to climate change. It further engaged and mobilized the people who will implement and sustain the solutions. Participants felt the solutions they came up with would markedly strengthen the hospital’s capacity and improve the quality and safety of care. Member checking interviews conducted after the VCA confirmed that workshops (in Stage 3 and 5) before discussions helped participants understand the project’s rationale, importance, and expectations.

Successes aside, any change management process will face its challenges. Here, we outline the main challenges we encountered in implementing this VCA in three domains: convincing and mobilizing staff, facilitating effective focus groups, and managing the data. We include strategies to overcome the challenges.

**Engaging and mobilizing the staff (as change agents).** Building a climate-resilient hospital is essentially a large change management activity that relies on people to initiate and maintain the change. For this, buy-in is critical, which is challenging in a setting of extreme, immediate needs and serious competing demands. There is an ongoing need to build awareness about the threat of climate change and to break down the technical terminology to create meaning for health staff so that they understand the direct links between their health work and the climate and environment they live in. Further, they understand what actions they can take to improve the situation, recognizing that some actions will have direct impacts on patients today and others have a longer lead time and possibly benefit future generations. However, throughout the process, the assessors observed increasing engagement and a sense of agency among hospital staff regarding sustainable health programming. This became an added, unexpected benefit of the VCA process.

**Facilitating effective focus groups for data collection.** Staff were comfortable with the quantitative audit process, but expressed concerns that formal Focus Group Discussions (FGDs) with audio recording would be unfamiliar in that setting, and would be a barrier to their engagement in the qualitative stage of the VCA. A regional anthropologist was hired to help develop an appropriate qualitative data collection approach, which consisted of facilitated discussion, using a storytelling approach with a pictorial recording of outputs (See Annex 2: Tree Diagram). Participants felt this was successful in achieving the engagement of a diverse range of interlocutors. Having a table-sized layout (map) of the hospital grounds helped teams visualize their situation and experiences, pointing to different parts of the hospital as the scenario progressed. Another difficulty was the language barrier: the reliance on interpreted communication meant that less could be covered than planned in the allotted time. Follow-up member checking interviews during a follow-up visit confirmed that participants felt the short timeframe restricted the depth of issue exploration, which could be overcome with extended time so that participants could fully grasp the tool and engage deeply. They also suggested supplementing focus groups with individual semi-structured interviews with key figures to gather additional insights.

**Managing the data.** The VCA approach poses the risk of generating a huge amount of data. An important challenge is planning data collection to get close to the outputs anticipated. There is a need to focus and plan on condensing and synthesizing the data. In Ngouri, reporting the outcomes of the FGD required consideration of what the team felt was most important, what to include, and what to leave out. While audio recording and note-taking were both discussed, the team opted to produce a tree diagram that outlined the root causes and solutions as a summary of the key issues discussed.

**Support post-VCA.** Towards the end of the climate VCA process, it became apparent that the production of a prioritized solution list would not be sufficient to enable the hospital team to progress towards the implementation of interventions. The CRESH team continued to work together to develop a comprehensive adaptation plan, with indicators and cost and human resource analysis. Funds were gradually raised through collaborative fundraising between ALIMA and CAA, and an extra field logistician was employed as well as a CAA regional health implementer based in Dakar, to visit every three months to provide training, support solution implementation, and carry out monitoring and evaluation. A monitoring framework was established, based on process indicators of solution implementation (integrated into standard activity reports of each relevant department), routine service delivery indicators (integrated into the hospital HIS, which is DHIS2), and outcome indicators (for which annual mini-VCAs are required). The first two categories of data are collected and reported monthly. The annual mini-VCA is completed in 1 day by the CAA regional support officer and consists of a repeat of the facility audit (stage 2), and one focus group discussion with staff.

## Discussion

To our knowledge, this is the first published climate change resilience building assessment implemented in a health facility in Chad. We summarize three key lessons learned. First, in very low resource settings, when the health system’s performance and emissions are low, we assumed decarbonization would not be a priority; however, local actors are much more positive about reducing environmental impact than expected. Second, there is a need to adapt the generic climate VCA approach to the specificities of each setting. Third, the list of solutions generated by the climate VCA process includes both general health system strengthening solutions and interventions that are more targeted to climate change-related health risks.

First, in very low-resource settings where vulnerability to climate change is very high and adaptive capacity is very low and where health facilities are fragile and emissions are low, one would assume decarbonization is not a priority at all. Yet, we found local actors were also motivated to reduce the environmental impact of healthcare and did not see this as an imposition or a separate goal. Environmental sustainability was viewed holistically, extending beyond carbon reduction to encompass the broader environmental impacts of healthcare, including efficient water use, improved waste management, recycling, and carbon reduction. These initiatives were framed as quality improvement efforts rather than solely decarbonization activities, which makes sense given the economic, social and health co-benefits that mitigation affords.

While existing frameworks often focus on decarbonization, CCA and low-carbon development are interconnected goals. The best CCA strategies would also reduce emissions and environmental impacts and the best environmentally sustainable activities would ideally also build resilience.

The fact that even in low-emitting, climate-vulnerable health facilities in Sub-Saharan Africa, health facility managers prioritize decarbonization alongside resilience-building highlights the complexity of balancing adaptation and decarbonization in low-income countries, where the goal would be to decouple future development from emissions. We found that there is an urgent need to build climate resilience, but this need not come at the expense of the environment.

Nevertheless, first things first and second things second. While Ngouri staff are very conscious and concerned about environmental sustainability, in the final prioritization activity, those initiatives that would have the greatest impact on climate resilience were prioritized before sustainability initiatives. While climate resilience initiatives mostly also increased sustainability, only one pure sustainability initiative was prioritized that has no direct impact on resilience, which was plastic recycling.

This finding raises the debate about the balance between mitigation and adaptation/resilience-building activities. Ideally, these activities should be synergistic, where resilience-building measures are inherently low-carbon, and mitigation efforts contribute to building resilience. Theoretically and practically, we found it better to avoid a stark juxtaposition between decarbonization and resilience because both are essential for improved health outcomes. Rather than fixating on measuring carbon footprints, the focus should be on implementing “no regret measures,” such as efficient water usage, rainwater harvesting, and effective clinical waste management, which simultaneously improve health, build resilience, and are low carbon.

There are different pathways for strengthening climate resilience and low-carbon sustainability in health systems depending on the baseline performance of the health system and the baseline emissions. If health system performance is low and health sector emissions are low, it makes sense to focus on climate resilience while adopting sustainable, low-carbon technologies. If health systems’ performance is mid-range and emissions are mid-range, it makes sense to strengthen both climate resilience and low-carbon sustainability. If performance is high and emissions are high, the focus should be on achieving net-zero emissions while continuing to promote climate resilience. Ultimately, the goal is to get high performance and low emissions in health systems [56].

VCAs aim to understand how various systems are susceptible to climate-related impacts and their capacity to adapt. VCAs have an evolving trajectory. Initially, VCAs were primarily focused on identifying vulnerabilities in sectors such as agriculture, water resources, and ecosystems and were used to develop National Adaptation Plans (NAPs). Over time, a better understanding of the relationship between climate change and health prompted an expansion of VCAs to include the health sector.

Over a decade ago, the WHO adapted VCAs to health systems [53]. The WHO has worked on developing guidelines, tools, and methodologies to assess the vulnerability and capacity of health systems in response to climate change impacts. These tools have been broadly applied across various regions to assess the vulnerability of health systems to climate change, considering factors such as extreme weather events, changing disease patterns, and health infrastructure resilience. VCAs have proven instrumental in identifying areas where health systems are most at risk and have guided the development of targeted adaptation strategies.

However, some health facilities are left out of these assessments and resilience-building activities. There is an opportunity for health facilities to take the initiative and conduct their own VCAs, specific to the hazards, exposure, and capacities of their particular environmental and social setting. In doing so, health facilities can develop a robust case for investment in mitigation and adaptation activities, develop the climate literacy of their workforce, boost engagement in change management projects that develop climate resilience and act as exemplars for other health facilities in their networks.

The history and background of VCAs provide the context for the second lesson learned—there is a need to adapt this generic climate VCA approach to different healthcare settings. A myriad of tools exists to assess the resilience of health facilities. However, we recognized early on that there is no single tool or a “one size fits all” approach to a VCA assessment of a health facility. This reflects the extremely diverse settings, populations, and facilities we are dealing with worldwide, as well as unique threats, needs, and priorities. In the case of Ngouri, the qualitative data collection step needed to be adapted for local circumstances; this sort of “configuration” should be anticipated every time the climate VCA is used. The current version of the climate VCA approach was developed for a hospital setting. If it were to be applied to a different health system level (such as primary healthcare), it would need to be adapted. Finally, to enable scale-up, the approach will need to be adjusted to be delivered by hospital leadership without the need for external climate change and health expertise or research expertise.

In the context of building the resilience of health facilities to climate change, we also learned that the endeavor involves more than narrowly defined “niche” projects directly addressing climate change hazards. While early warning systems for extreme weather events are important components, a holistic approach is needed, especially in low-resource settings. This approach entails developing the baseline ability of health facilities to consistently deliver quality and accessible healthcare services. For instance, the reliability of access to energy, a safe and sufficient water supply, and an adequate health workforce are fundamental prerequisites. In the absence of these foundational elements, the effectiveness of early warning systems becomes compromised.

This leads us to the third main lesson learned—the list of solutions includes both general health systems strengthening solutions and targeted climate change-related health risk interventions. Therefore, the discussion around solutions should encompass both general health systems strengthening measures and targeted interventions addressing climate change-related health risks. Strengthening components such as the blood bank and supply, which may not be explicitly climate change-focused, reflect the overlapping nature of the risks resulting from specific hazards, and the fact that a given solution may reduce risk from multiple hazards. This aligns with the endorsed “all hazards” approach to risk reduction and emergency preparedness advocated by entities like the WHO and other stakeholders. Embracing a comprehensive strategy acknowledges that building resilience is a multifaceted challenge that requires addressing both specific climate change hazards and reinforcing the broader health system [40].

## Conclusion

The facility-adapted climate VCA provides a practical and replicable approach to assessing climate vulnerabilities and capacities in similar settings. The results of the climate VCA emphasized the significant health risks posed by climate change, including an increased risk of malnutrition and malaria, and revealed that even in the least developed settings, sustainable solutions are a local priority. The 22 climate solutions identified included anticipatory planning, community engagement, and improved waste management, which together constitute a strategic approach for building climate resilience in Ngouri Hospital and provide a blueprint that could be adapted to similar healthcare facilities in the Sahel region and beyond.

Health sector leaders, policymakers, researchers and practitioners might like to integrate climate considerations into existing tools, checklists and resources that assess health systems’ strength, capacity and resilience. The lessons learned in the climate VCA process highlight the significance of community involvement, interdisciplinary collaboration, and effective data management for carrying out such a participatory process. This study contributes to the broader goal of aligning healthcare systems with global sustainability objectives while ensuring access to quality healthcare in the context of a changing climate.

## Footnotes

1 The Climate Action Accelerator, Climate Vulnerability and Adaptation Toolkit is available here: https://climateactionaccelerator.org/climate-vca/

2 The generic solutions list can be found within the CAA VCA Toolkit available here: https://climateactionaccelerator.org/climate-vca/

## Role of Authors

All authors had access to the data and contributed to the writing of the manuscript. Conceptualization and design: PNS, KJ, MM. Data collection: MM, DTZ, ARD, MT. Data analysis: PNS, KJ, MM. Manuscript preparation: PNS, ZH. Review and editing: All authors.

## Additional Files

The additional files for this article can be found as follows:

Supplementary Annex 1. 35 Potential Solutions.

Supplementary Annex 2. Tree Diagram. Output of the Focus Group Discussions. Using a tabletop exercise, the groups used a tree diagram to capture climate hazards (roots: gray), climate impacts (branches: yellow) and solutions to mitigate these risks (branches: green). Below is the Tree Diagram from the District Health Administrators focus group.

Supplementary Annex 3. Climate Information sources (For stage 1 and 2).
