# Supplementary material for: Building Climate Resilience in Health Systems: A Climate Vulnerability and Capacity Assessment in a rural hospital in Chad
Source: Ann Glob Health. 2025 Aug 19;91(1):50. doi: 10.5334/aogh.4743 (PMC12372681; doi:10.5334/aogh.4743)
Supplement: Supplementary Annex 1. — 35 Potential Solutions. [file agh-91-1-4743-s1.pdf]

ANNEX 1

35 Potential Solutions

Solution No.	Module	Solution description
1	Infrastructure	Reduce overall internal temperatures through painting roofs with highly reflective paint.
2	Infrastructure	Increase shade and cool areas for staff rest. Provide drinking water and food for staff.
3	Infrastructure	Recondition / repaint internal surfaces of hospital to improve infection control.
4	Infrastructure	Improve ventilation through ventilation chimneys and 'louvre' windows / shutters.
5	Infrastructure	Additional multifunctional structure to increase bed capacity for peaks.
6	Infrastructure	Improve O2 management to ensure resilience for peaks (efficient concentrators / bridging system)
7	Infrastructure	Supply chain analysis and all hazards resilience strengthening.
8	Energy	Develop and implement energy management protocol to reduce consumption.
9	Energy	Improve energy infrastructure to ensure resilience for peaks and to stabilise temps. in ICU / pharmacy. Extend solar system; build a cabin to better house batteries with an appropriate bridging system; movement sensors to reduce lighting use.
10	Wash and waste	Adapt medical waste management processes, malaria protocols and severe acute malnutrition protocols for peaks, to prevent service overload.
11	Wash and waste	Strengthen medical waste infrastructure through a communal waste zone to improve resilience during peaks.
12	Wash and waste	Increase water security by collecting rainwater for laundry to reduce demand.
13	Wash and waste	Evaluate possibilities for waste recycling (e.g., plastics) at Ndjamena.
14	Workforce	Health workforce planning for peaks of service demand (aka peaks) based on anticipatory data. Include rest periods during heatwaves.

15	Workforce	Staff education for self-care and sustainable healthcare, energy management, waste management. Include the effects of heat on patients and the changing distribution of infectious disease.
16	Workforce	Establish a hygiene committee for the whole hospital.
17	Workforce	Promote or develop local / hospital level disaster management committee.
18	Programs	Anticipatory systems/seasonal calendars used to plan buffer stocks and for hospital planning more broadly.
19	Programs	Bed net distribution and indoor residual spraying.
20	Programs	Free hospital care for children.
21	Programs	Affordable referral system (e.g. - free ambulance service, including boat transfer) that addresses main community barriers to accessing care.
22	Programs	Community sensitisation (malaria, NCD, diarrhoea etc.)
23	Programs	Supplementary feeding program 3 months before malarial peaks / once food crisis threshold met.
24	Programs	Routine community surveillance (malnutrition, malaria, meningitis, water-borne diseases).
25	Programs	Review and strengthen facility contingency planning (EPREP) to cover all hazards including climate related / pandemics.
26	Programs	Community based interventions (e.g., mothers screening for malnutrition and providing nutrition support, vaccine campaigns).
27	Programs	Reinforce blood bank including sensitisation of donor recruitment and supply of testing reagents.
28	Programs	Establish a malaria vaccination program.
29	Programs	Integrate infectious disease innovations: meningitis chemoprophylaxis, rotavirus / oral cholera vaccine.
30	Programs	Innovative malnutrition approaches to reduce incidence of SAM (e.g., simplifying protocols, home monitoring of MUAC)
31	Programs	Primary Care interventions: reinforce training / staffing / stocks / service organisation e.g., Observation room
32	Programs	Patient self-care training and medication stocks in anticipation of service disruption.

33	Leadership Coordination	Advocating / supporting national DRR work, climate smart agriculture, social protection programs, irrigation and water storage, national disease control programs.
34	Leadership Coordination	Monitoring and evaluation using appropriate indicators to improve / adjust choice and implementation of interventions
35	Leadership Coordination	Integrate with ALIMA environmental roadmap and other country initiatives to ensure sustainable financing
