# Supplementary material for: Building Climate Resilience in Health Systems: A Climate Vulnerability and Capacity Assessment in a rural hospital in Chad
Source: Ann Glob Health. 2025 Aug 19;91(1):50. doi: 10.5334/aogh.4743 (PMC12372681; doi:10.5334/aogh.4743)

ANNEX 2

Tree Diagram. Output of the Focus Group Discussions. Using a table top exercise, the groups used a tree diagram to capture climate hazards (roots: grey), climate impacts (branches: yellow) and solutions to mitigate these risks (branches: green). Below is the Tree Diagram from the District Health Administrators focus group.

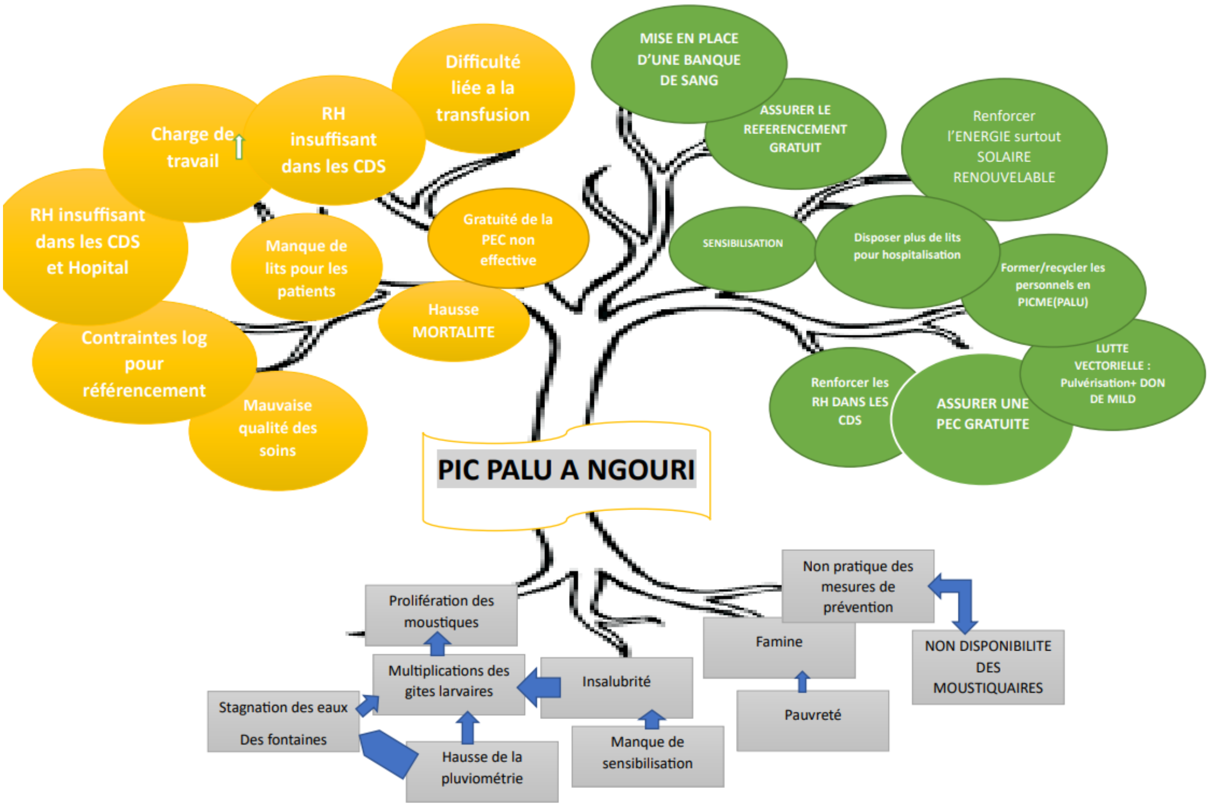

Supplement: Supplementary Annex 2. — Tree Diagram. Output of the Focus Group Discussions. Using a tabletop exercise, the groups used a tree diagram to capture climate hazards (roots: gray), climate impacts (branches: yellow) and solutions to mitigate these risks (branches: green). Below is the Tree Diagram from the District Health Administrators focus group. [file agh-91-1-4743-s2.pdf]
