# Supplementary material for: Building Climate Resilience in Health Systems: A Climate Vulnerability and Capacity Assessment in a rural hospital in Chad
Source: Ann Glob Health. 2025 Aug 19;91(1):50. doi: 10.5334/aogh.4743 (PMC12372681; doi:10.5334/aogh.4743)
Supplement: Supplementary Annex 3. — Climate Information sources (For stage 1 and 2). [file agh-91-1-4743-s3.pdf]

ANNEX 3

Climate Information sources (For stage 1 and 2).

Data source type	Model	Source	Products
Seasonal Climate Forecast Model	North American Multi-model Ensemble Project (NMME)	International Research Institute (IRI) for Climate and Society. Columbia Climate School	Tertile summary maps, Flexible seasonal maps, Verification plots.
Climate Change Information Services	Copernicus Climate Change Service (C3S) multi-system ensemble	Copernicus	Ensemble mean anomaly maps, Tertile summary maps, Extreme 20th percentile maps, Verification plots.
Probabilistic Multi-model Ensemble (MME)	World Met. Org (WMO) Centre for Long-Range Forecast	WMO	Tertile summary maps.
Global Ensemble System	Global Ensemble Prediction System (GEPS)	Climate Engine	Low resolution (55km2) cumulative rainfall and avg. temps with an online visualising tool.
Weather Forecast Tool	European Centre for Medium Range Weather Forecasts (ECWMF)	ECMWF	Rainfall, temp, wind, air pressure online visualising tool.
